# Assessment of Challenging Behavior Exhibited by People with Intellectual and Developmental Disabilities: A Systematic Review

**DOI:** 10.3390/ijerph19148701

**Published:** 2022-07-17

**Authors:** Juliana Reyes-Martín, David Simó-Pinatella, Josep Font-Roura

**Affiliations:** 1 Facultat de Psicologia, Ciències de l’Educació i de l’Esport, Blanquerna, Ramon Llull University, 08022 Barcelona, Spain; davidsp@blanquerna.url.edu; 2 Fundació Vallparadís, Mutua Terrassa, 08221 Barcelona, Spain; 3 CPT Estel, Vic, 08500 Barcelona, Spain; fontplanella@gmail.com

**Keywords:** assessment, scales, challenging behavior, intellectual and developmental disabilities, systematic review

## Abstract

The assessment of challenging behavior exhibited by people with intellectual and developmental disabilities is essential for the planning of prevention and intervention programs. This review aimed to identify and analyze the standardized instruments that exclusively focus on the assessment of challenging behavior. We identified and organized 141 articles into four categories: original instrument studies, validation studies, relational studies, and intervention studies. The results identified 24 instruments that generally show high-quality psychometric properties and other utilities beyond the observation of the presence of challenging behavior and diagnostic categorization. Age, level of adaptive behavior, disability, presence of autism spectrum disorder, and medication are some of the variables that were found to be possibly related to the occurrence of challenging behavior. Additionally, the results suggest that interventions focused on supporting positive behavior or providing training on behavior to professionals and caregivers significantly reduced the occurrence of these behaviors. Instruments that help us to understand and measure the challenging behavior exhibited by people with intellectual and developmental disabilities are essential for the design of effective evaluation and intervention protocols.

## 1. Introduction

Challenging behavior (CB) [1] exhibited by people with intellectual and developmental disabilities (IDDs), such as aggressive, stereotypic, self-injurious, and disruptive behaviors, is a cause for concern. The frequency, severity, duration, and resistance to treatment of CB are problematic for families and professionals [2]. CB influences personal and emotional well-being, and can limit access to experiences and opportunities [1].

The prevalence of CB exhibited by people with IDDs is highly variable according to the population studied, the behaviors that are prioritized, and the procedures and methodological instruments that are used [3,4,5]. One of the most recent reviews [6], which involved 20 studies, analyzed the prevalence of CB in school-aged individuals with disabilities. The results indicated that the overall prevalence rates ranged from 48% to 60% in children with IDDs, while studies measuring CB in children with autism recorded a level of 90%. The most frequent CBs are aggression and self-harming behaviors. Despite the limitations inherent in prevalence studies, the magnitude of behavioral difficulties is a cause for concern, making it imperative to develop and use assessment and intervention procedures that have been sufficiently tested and which are supported by strong empirical evidence. Thus, the evaluation of CB is an essential preventive measure to reduce the impact that the behavior has on the individual and those who live in their environment. Given the importance of evaluation systems in the treatment of CB, several studies have been carried out in recent years to assess the use and degree of effectiveness of different procedures [7,8].

CB exhibited by people with IDDs has been mainly assessed using two approaches: direct and indirect descriptive procedures. Direct descriptive procedures use direct observation of the person in their natural environment [9]. However, descriptive procedures have a number of limitations, such as difficulty in understanding and interpreting environmental variables, as well as the amount of time required [10]. On the other hand, indirect descriptive systems require the views of informants (either the individual concerned or significant people in their environment) about the assessed behavior, which are collected through interviews, scales, checklists, and questionnaires [11,12,13]. Although the information gathered using indirect instruments may not be as accurate as that obtained using direct measures, their general ease of use, the time taken, and the training required are aspects worth bearing in mind when considering the evaluation of CB [13,14].

Although there is no agreement among researchers and practitioners, the use of standardized measures could be helpful in the assessment of CB and design of intervention plans. In general, indirect measures meet most of the objectives of a measurement instrument: evaluation, diagnosis, screening, and monitoring of progress [15]. In other words, they allow professionals to collect information about the type and severity of the CB that a person engages in, to identify and understand in greater depth the behaviors and their main causes, and to monitor both the progress of the individual and the effectiveness of the intervention that has been carried out.

Authors of recent research have made efforts to systematically review the tools used to assess CB. For instance, a review that aimed to assess the evidence for the validity of the criteria of CB scales published since 2000 identified and analyzed 12 scales used to evaluate the CB of people with IDDs [16]. The conclusions of this study suggested that the criterion validity of the scales was not satisfactory and more attention needed to be given to the validity of the content. Similarly, a recent review [17] aimed to identify relevant measures (psychometric properties) for the assessment of mental health disorders among children and young people with intellectual disabilities. The results of the study identified ten instruments that could be used to assess mental health disorders. However, these reviews all focused on the study of the psychometric properties of these tests. To the best of our knowledge, no study has focused on the evidence relating to the instruments, i.e., exploring how they have been used by researchers. Assessment procedures should help researchers and practitioners to understand the nature of the behavior, as well as to design appropriate interventions [18].

### The Present Study

The aim of our study was to explore the state of the evaluation of the CB exhibited by people with IDDs. To this end, the main objective was to identify standardized instruments that focus solely on the CB presented by people with IDDs and to analyze their rigor and usefulness. Thus, the specific objectives that emerged were the following: (a) to study the psychometric characteristics and properties of these instruments, (b) to explore the relationship between the CB assessed using these instruments and the variables that may be related, and (c) to identify the evidence provided by the literature related to the psychoeducational interventions carried out based on the results of evaluations of the CB.

## 2. Method

A systematic review of standardized instruments used to assess the CB exhibited by people with IDDs was conducted, taking into consideration the recommendations of the Cochrane Collaboration [19] and the PRISMA Statements [20] (see Appendix A for Search strategy through electronic databases).

### 2.1. Information Sources and Eligibility Criteria

A literature review was carried out using the bibliographic databases PsycINFO, Medline (PubMed), Web of Science (WoS), and Education Resources Information Center (ERIC). The search strategy was peer-reviewed, and it considered records published from 2000 to March 2022.

The database search was performed by combining the following keywords and key phrases: intellectual and developmental disability AND challenging behavior OR problem behavior AND evaluation OR assessment OR questionnaire OR inventory OR scale. The terms had to appear in the title and/or summary. The complete search strategy for the PubMed database is presented in Appendix A. The last search was carried out in April 2022.

The eligibility criteria focused on peer-reviewed articles that (a) were published between 2000 and 2022, (b) targeted people with IDDs (in studies with multiple groups, those that included at least one group with people diagnosed with IDDs were taken into account), (c) aimed to design and develop a specific tool to detect CB, to adapt it and/or to evaluate its psychometric properties, (d) examined the unidirectional or bidirectional relationship between CB and other variables, and (e) were empirical studies that measured the results of a psychoeducational intervention for addressing CB.

Considering these inclusion criteria, we excluded from this review those studies that (a) evaluated CB in people without IDDs (e.g., [21]), (b) examined challenging behavior using specific subscales of broader tools (measures of adaptive behavior and/or mental health containing specific subscales of CB (e.g., [22,23]), (c) did not use standardized tools (e.g., [24,25]), and (d) were theoretical studies or reviews of the literature (e.g., [9,26]).

### 2.2. Search Strategy and Study Selection

The results obtained from the bibliographic databases identified 1379 records (PubMed, 269; PsycoINFO, 384; WoS, 342; and ERIC, 384).

The results obtained were merged using Mendeley reference management software, and duplicates were automatically removed. Once 347 duplicates were eliminated, a list of titles was compiled. In parallel, the first and second authors independently assessed and identified the potential relevance of the 1032 articles by examining titles and abstracts following a checklist that included prespecified eligibility criteria. A total of 630 articles were removed as they did not meet the predetermined inclusion criteria.

**Figure A2:**
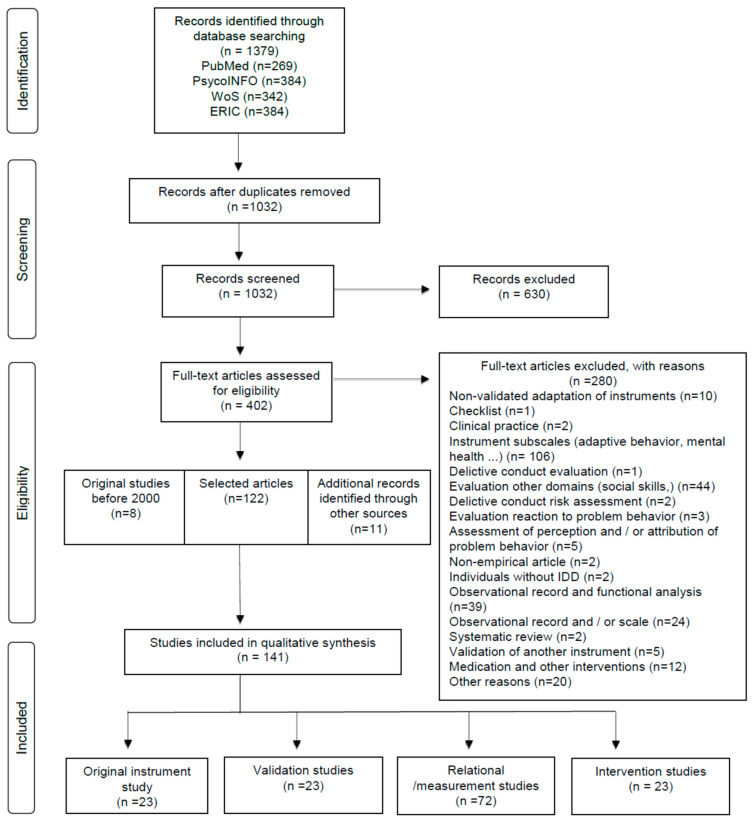
PRISMA Flow diagram.

Both authors carried out a full-text reading of the 402 articles to verify that they met the eligibility criteria. Disagreements were resolved by consensus, and where this was not possible, the text was reread and/or the third author was consulted. As a result of the application of the exclusion criteria, 122 articles were selected, and 280 were excluded. Two additional searches were then conducted. First, original instruments published before 2000 were identified during the search procedure. Second, an independent search was performed for each identified instrument to detect any articles that could have been missed during the search procedure. A total of 19 articles were added to the literature review (8 and 11 for each additional search, respectively). In conclusion, a total of 141 articles were included in this review, as shown in Figure A2 (Appendix B).

### 2.3. Data Analysis

The first and second authors conducted a full-text reading of the selected studies. The included studies were classified into four categories according to the objective of the study (original instrument, validation, relational and/or measurement, or intervention study). In line with the Cochrane recommendations, a summary sheet per category was developed for data extraction:

- For original studies related to the instrument, the data extracted were tool, abbreviation, original reference, specific characteristics, purpose and approach, method of application, number of items, domain of behavior, type of evaluation (frequency, severity, intensity, etc.), target group, scoring method, and psychometric properties.
- The validation category included validation studies with instrument-independent samples. Aspects related to the tool, reference, country, number of participants, age range, objective, and psychometric properties were extracted.
- In the relational and/or measurement studies, CB was correlated with other variables of interest. For this purpose, the data extracted were instrument, reference, number of participants, age range, associated variables, and the association index.
- CB was considered the dependent variable for the intervention studies. The information extracted was tool, reference, number of participants, age range, level of intellectual disability, diagnosis of autism spectrum disorder, design, components of the intervention, and results.

### 2.4. Risk of Bias and Applicability

We used the Cochrane risk of bias tool [27] for risk of bias assessment. Two review authors from the research team independently assessed and made an argument-based judgment (low, high, or unclear) in the seven domains proposed by the Cochrane tool for each of the studies: random sequence generation, allocation concealment, blinding of participants and staff, blinding of outcome assessors, incomplete outcome data, selective reporting of results, and other sources of bias. Disagreements between the two researchers were resolved via discussion or by involving a third expert to make a judgment about the risk.

## 3. Results

The 141 studies included in this review were categorized according to whether they were original articles on instrument development (*n* = 23), validation (*n* = 23), relational and/or measurement (*n* = 72), or intervention (*n* = 23). It is important to mention that ten studies were categorized as belonging to more than one category. Specifically, six studies [28,29,30,31,32,33] were grouped into the categories of relational and/or measurement and validation studies, and two studies [34,35] were grouped into the categories of relational and intervention studies.

### 3.1. Original Instrument Studies (n = 23)

Out of the 141 selected articles, our systematic review identified 23 in which the objective was to develop and validate a specific instrument for evaluating CB [36,37,38,39,40,41,42,43,44,45,46,47,48,49,50,51,52,53,54,55,56,57,58]. One study developed and validated two instruments: the Eyberg Child Behavior Inventory (ECBI) and the Sutter–Eyberg Student Behaviour Inventory–Revised (SESBI-R). The main characteristics of the 24 instruments for the evaluation of CB can be found in Table 1, together with the validity and reliability scores of the original instrument.

Eight instruments were developed before the 2000s (Aberrant Behavior Checklist, ABC-R; Aberrant Behavior Checklist—Community, ABC-C; Modified Overt Aggression Scale, MOAS; Self-Injury Trauma scale, SIT Scale; Checklist of Challenging Behavior, CCB; Stereotyped Behavior Scale, SBS; ECBI; and SESBI-R). All instruments except the Behavior Problems Inventory for People with Profound Intellectual and Multiple Disabilities (BPI-PIMD) [53] were originally validated in English. The instruments identified in this review addressed different populations. Specifically, 12 instruments focused on the assessment of the CB exhibited by children and adults with IDDs. Of these 12, 10 focused on this population without a specific level of IDD or diagnosis (ABC; ABC-C; Behavior Problem Inventory, BPI-O1; Behavior Problem Inventory—Short, BPI-S; Behavior and Sensory Interests Questionnaire, BSIQ; Challenging Behavior Interview, CBI; CCB; Problem Behavior Checklist, PBCL; Repetitive Behavior Questionnaire, RBQ; and Repetitive Behavior Scale—Revised, RBS-R), whereas one (BPI-PIMD [53]), was a specific adaptation of BPI-01 [55] for children and adults with profound and multiple IDDs; and the Challenging Behavior Questionnaire (CBQ) [44] was specific to people with Cornelia de Lange syndrome. Seven instruments were identified that were designed for adults with IDDs. Five out of the seven were intended for adults with IDDs without a specific level or diagnosis (Adult Scale of Hostility and Aggression: Reactive–Proactive, A-SHARP; Institute for Basic Research—Modified Overt Aggression Scale, IBR-MOAS; MOAS; SBS; and Stereotyped Behavior Scale Revised, SBS-R), whereas one (MOAS [46]), although originally designed for populations with psychiatric disorders, was validated and used for adults with IDDs [59]. On the other hand, the Autism Spectrum Disorder—Behavior Problems for Adults (ASD-BPA) instrument [49], although also designed for adults, was specific to people with autism spectrum disorder and IDDs. Two instruments focused on the CB exhibited by children with IDDs without a specific level or diagnosis: the Children’s Scale of Hostility and Aggression: Reactive–Proactive (C-SHARP) [41] and Behavior Problems Inventory for School (BPI-S-SCHOOL) [51]; and one, the Autism Spectrum Disorder—Problem Behaviors Child (ASD-PBC) [48], was designed for children with autism spectrum disorder and IDDs. Finally, there were three instruments that were not originally designed for people with IDDs, although they were adapted and validated for this population; two for children (ECBI and SESBI-R) and one for children and adults (SIT Scale [45]).

Of the 24 instruments considered, 16 explored different types of CBs (ABC, ABC-C, ASD-BPA, ASD-PBC, BPI-01, BPI-S, PIMD BPI-PIMD, BPI-S-SCHOOL, CBI, CBQ, CCB, ECBI, SESBI-R, PBCL, IBR-MOAS, and MOAS). The other eight instruments focused on one type of CB (A-SHARP, C-SHARP, BSIQ, RBS-R; RBQ, SIT Scale, SBS, and SBS-R).

Most of the instruments were rating scales (ABC, ABC-C, A-SHARP, C-SHARP, ASD-BPA, ASD-PBC, BPI-01, BPI-S, BPI-PIMD, BPI-S-SCHOOL, CCB, ECBI, SESBI-R, IBR-MOAS, MOAS, PBCL, RBS-R, SIT Scale, SBS, and SBS-R), whereas three were questionnaires (CBI, CBQ, and RBQ) and one was a semi-structured interview (BSIQ). All of the information was collected based on informants who were usually family, caregivers, or reference professionals, i.e., people who were familiar with the person who engaged in the behavior. When analyzing the way professionals rated the assessed behaviors, most of the identified instruments (ABC-R, ABC-C, ASD-BPA, ASD-PBC, BPI-01, BPI-S, BPI-PIMD, BPI-S-SCHOOL, ECBI, SESBI-R, IBR-MOAS, MOAS, and SBS-R) used two Likert rating scales to identify the frequency and severity of the behavior. Other instruments, in addition to frequency and severity, explored other variables when assessing CB, including the following: whether the behavior was proactive or reactive (A-SHARP and C-SHARP); when it began and its duration (BSIQ [42]); the difficulty of managing the behavior and the strategies used (CCB [43]); the duration and effects of the behavior on the person engaging in the behavior and its effects on others (CBI); and the type and location of injuries (SIT scale). Three instruments solely explored the frequency of the behavior (CBQ, RBQ, and SBS), and two focused on its severity (PBCL and RBS-R).

Based on different inter-reliability standards [60,61,62,63], internal consistency was excellent for SBS-R, ASD-PBC, ECBI, SESBI-R, and ASD-BPA; good to excellent for ABC, ABC-C, BPI-01, BPI-S, BPI-S-SCHOOL, and A-SHARP; good for RBQ, BPI-PIMD, SBS, and MOAS; and from regular to good for BSIQ and C-SHARP. However, the CCB [43] was considered unreliable as a measure to monitor change. Internal consistency was not estimated for five instruments (PBCL, IBR-MOAS, CBI, CBQ, and RBS-R). On the other hand, based on international standards [64,65,66], the level of reliability among evaluators was considered excellent for SBS-R, MOAS, BPI-01, PBCL, and BSIQ; from good to excellent for A-SHARP and the C-SHARP problem subscale; good for SESBI-R, RBQ, RBS-R, ABC, and ABC-C; from regular to excellent for ASD-PBC; from regular to good for CBI and IBR-MOAS; between poor and excellent for the provocation subscale of C-SHARP; and between poor and good for ASD-BPA. For the rest of the instruments, it was not evaluated. With regard to the reliability test, the retest was considered excellent for ABC, ABC-C, SBS, SBS-R, MOAS, CBI, and BSIQ; good to excellent for ASD-PBC, BPI-01, BPI-PIMD, and IBR-MOAS; good for SESBI-R, RBQ, and RBS-R; and between poor and excellent for ASD-BPA. For the rest of the instruments, it was not calculated.

### 3.2. Validation Studies (n = 23)

With regard to the studies that continued to explore the psychometric properties of the selected instruments or validated them with other populations, a total of 23 articles involving 9 instruments were identified (see Table 2).

Four studies explored the psychometric properties of more than one instrument. The most studied instruments were BPI-01 [31,68,69,72,73,74,75,76,77,78,79,80,81], BPI-S [82,83,84,85], and RBS-R [75,86,87,88]. In addition, the properties of ABC-C [70,71], ABC [67,68], MOAS [59], and A-Sharp [72] were studied.

In relation to BPI-01, five studies re-examined its psychometric properties with different samples that involved children and/or adolescents [68,80], adults [76,81], or both children and adults [69]. The convergent validity of the BPI-01 was explored for ABC [69], A-SHARP and ABC [72]. Psychometric properties were also explored using the theory of response to the instrument, taking account of possible bias according to sex, using both BPI-01 and BPI-S [82]. Moreover, BPI-01 was translated into and validated in several different languages: Chinese [73], Korean [77], Swedish [78], Romanian [79], Brazilian [74], and Dutch [75].

The BPI-S was another instrument that was translated into and validated in different languages: Japanese [83] and French [85]. This instrument was also examined using an independent sample that involved both children and adults [84].

Three other instruments had evidence from different settings and/or samples. First, the RBS-R had standardized scores according to diagnostic group using a sample of children and adults in Spain [88], and its psychometric properties were re-examined in a sample of children with IDDs in Peru [68]. This instrument was translated into and validated in Japanese [86] and German [87]. Second, the psychometric properties of the ABC were re-examined in a sample of children with IDDs in Peru [68] and the construct validity of the Norwegian version was tested using a sample of children and adolescents [67]. The community version of the ABC was included in two studies: one that examined the factorial validity of the original English version of the ABC-C in a sample of children and adolescents [70] and another that standardized and examined the psychometric properties of the German version of the ABC-C [71]. Finally, the reliability of the MOAS in people with IDDs was also explored [59].

### 3.3. Relational Studies (n = 72)

Of the articles identified in this review, a total of 72 were identified as relational or measurement studies (see Table 3). Six studies, although categorized as relational and/or measurement, overlapped with the validation category [28,29,30,32,33,68].

As shown in Table 3, most of the relational studies used a version of the BPI [29,31,122,123,124,125,126,127,128,129,130,131,132,133,134,135,136,137,138,139,140,141,142], the ABC [94,95,96,97,98,99,100,101], or the ABC-C [33,105,106,107,108,109,110,111]. The other instruments used for these studies were ASD-BPA [115,116,117,118,119], CCB [30,147,148,149,150,151], MOAS [153,154,155], RBS-S [157,158,159], IBR-MOAS [152], RBQ [156], and A-SHARP [121]. Thirteen studies used different combinations of the aforementioned instruments [28,32,102,103,104,112,113,120,143,144,145,146].

The number of participants included in these studies was 18,350 (10,605 of whom were males). Two studies did not report the sex of the participants [119,158], and one provided only approximate data [123]. Three studies reported the number and sociodemographic characteristics of the support staff but not of the participants with IDDs [133,149,150]. The ages of the participants in the samples ranged from 4 months to 93 years.

Different variables were examined in relation to CB. Table 3 identifies the variables that were studied in each of the selected articles.

#### 3.3.1. Age, Sex, and Race (*n* = 14)

Age was revealed as a factor differentially associated with CB [137]. For example, the results of one study suggested that CB usually appears before one year of age [104] and that severity decreases as age increases [151]. However, another study found no correlation between age and CB [131].

With regard to changes in pattern with increasing age, two studies found significant improvements in most typologies [98,106]. Seven studies detected a general pattern of stability, although with significant differences in specific topographies [33,94,100,107,108,143,157]. Hyperactivity and noncompliance showed significant reductions between childhood and early adolescence [94] and between adolescence and early adult life [100]. In addition to improvements in hyperactivity and noncompliance, reductions in irritability and noncompliance were also detected in a sample with IDDs and *Fragile X syndrome* [108]. With respect to self-injurious and stereotypic behavior, younger age was revealed as a risk factor for self-injurious behavior [107], and older age was revealed as a risk factor for stereotypic behavior [157]. Similarly, Schmidt et al. (2013) [33] detected that younger children typically score higher on certain ABC-C subscales.

Sex was revealed as a variable associated with CB [107,108,137]. For example, Hustyi et al. (2014) [108] used a sample of people with *Fragile X syndrome*, a sex-linked genetic syndrome. However, three studies failed to identify a relationship between sex and CB [95,131,157]. Specifically, Folch’s study (2018) [107] explored the association with self-injurious behavior, and Hoch et al. (2016) [157] explored the association with self-injurious and stereotypic behavior.

No significant differences were found between race and CB, although a significant interaction between autism spectrum disorder and race was detected in relation to CB [115].

#### 3.3.2. Level of IDD, Adaptive Behavior, Language, and Support Needs (*n* = 18)

Deficits in adaptive behavior were associated with CB [95,97,106]. According to the results of one study, control of the autism spectrum disorder variable moderated the effect of adaptive behavior, and the control of adaptive behavior moderated the impact of autism spectrum disorder symptomatology on CB [97].

The level of intellectual impairment was also systematically linked to the presence, severity, and topography of CB [29,30,31,99,100,107,124,129,135,138,145,158]. According to the results of three studies [31,124,158], the differences were significant only for stereotypic and self-injurious behaviors [31]. However, the results from other studies suggest that these findings were relevant for aggressive behavior [135] and contact behavior [30].

The presence of CB was positively correlated with the level of language impairment [100,118,138]. Specifically, language impairment was revealed as a risk marker for self-injurious behavior [100,118]. Additionally, correlation between CB and sensory hypersensitivity was also detected [129]. Moreover, the severity of these behaviors was also related to the attention provided by professionals or support staff [97,136], in that it was reported that people with IDDs who exhibited CB were likely to receive more attention from support staff [97].

#### 3.3.3. Quality of Life, Participation, and Life Events (*n* = 5)

According to one study, quality of life is negatively correlated with the presence of CB [99]. Discrete significant associations were detected between CB and social and community activities and household participation [97]. People with IDDs were more frequently exposed to negative life events [140], and these events were significantly related to the presence of CB [110,140], particularly aggressive behavior [153].

#### 3.3.4. Autism Spectrum Disorder (*n* = 17)

All of the studies that explored the relationship between autism spectrum disorder and CB concluded that people with IDDs and autism spectrum disorder exhibited significantly more CBs than people with IDDs alone [32,95,99,100,103,107,115,116,117,119,120,126,129,141,143,145]. According to Sappok et al. (2014), autism was the only mental disorder associated with CB in general. Likewise, it was noted that children with IDDs and autism spectrum disorder showed significantly more CBs than people with IDDs and Down’s syndrome or with atypical development [137]. Three studies concluded that the severity of autism spectrum disorder symptomatology correlated positively with the number and intensity of CBs [32,100,120]. In fact, Rattaz et al. (2018) suggested that the severity of autistic symptomatology was the main risk factor for the development of CB. Regarding specific topography, people with IDDs and autism usually showed significantly higher levels of self-injurious [32,107,118] and stereotypic behavior [32]. Finally, certain dimensions of quality of life were identified as mediating factors between autism spectrum disorder and CB.

#### 3.3.5. Epilepsy (*n* = 4)

The results of three studies suggested that comorbid epilepsy did not appear to be related to CB [106,107,135]. However, Smith and Matson’s study (2010) did find significant differences in the topography of aggression and/or destruction and stereotyped behavior.

#### 3.3.6. Genetic Syndrome and Others (*n* = 15)

Diagnosis is a variable associated with CB [137]. The results of the four studies that explored the relationship between CB and Down’s syndrome suggested that people with Down’s syndrome tended to have significantly fewer CBs than people with other diagnoses [109,126,129,145]. For example, children with autism spectrum disorder were rated as exhibiting more CBs than children with Down’s syndrome and other IDDs of mixed etiology [126].

The association between *Fragile X syndrome* and CB, as well as other genetic syndromes, was also explored in the literature. For example, heterogeneity in relation to repetitive behaviors between different genetic syndromes was identified. People with *Fragile X syndrome* exhibited significantly more problematic repetitive behaviors than people with Angelman’s, Cornelia de Lange, Cri-du-Chat, Prader–Willi, Lowe, and Smith–Magenis syndromes [156]. Moreover, the results seemed to suggest that children with *Fragile X syndrome* had restricted interests, more severe sensory behaviors, and less problematic self-harm [158]. On the contrary, the presence of CB was identified among people with Phelan–McDermid syndrome [111], but no significant differences in rates of stereotypic, self-injurious, and equality behaviors were found among people with *Fragile X syndrome* and autism spectrum disorder [159].

The results reported by DiStefano et al. (2020) indicated that children with isodicentric duplications (dup15q syndrome) tended to present significantly more challenging behaviors. There was no significant association between Cornelia de Lange and self-injurious behavior [112]. With regard to Smith–Magenis syndrome, the results suggested a significant association between this syndrome and CB, which was reported to be more frequent and serious [122].

The results exploring the association between CB and tuberous sclerosis were contradictory. For instance, although high rates of CB were identified in children with tuberous sclerosis, these rates were not significantly higher than those in children with other syndromes (e.g., Down’s syndrome) [144]. In contrast, in a sample of adults with tuberous sclerosis, rates of self-injurious behavior and aggression were significantly higher than in those with Down’s syndrome [146].

Finally, in the case of Pallister–Killian syndrome, one study suggested that stereotypic, aggressive, and self-injurious behaviors were frequent, but that these were mediated by other variables such as the level of adaptive behavior, the sensory profile, and/or sleep impairment [142].

#### 3.3.7. Other Medical Issues (*n* = 3)

Evidence suggests that gastrointestinal problems had a positive association with the presence of stereotypic behavior [100], and dental pain with self-injurious behavior [107]. Visual impairment was also associated with the presence of aggressive, self-injurious, and stereotypic behavior [137].

#### 3.3.8. Psychiatric Disorders, Sleep Disorders, and Psychopharmacies (*n* = 16)

People with IDDs and psychiatric disorders tended to have higher levels of CB [29,96,107,113,125,127,134,139,152,153,154,155,158]. This association was most prominent in people with severe IDDs [96,139], and with IDDs and psychiatric morbidity [154]. Moreover, aggressive behavior was associated with dual pathology [155], and psychiatric disorders could be considered as potential risk factors for self-injurious behavior [107].

Although no specific, significant association was established with a particular psychiatric disorder [125], deficits in impulse control, emotional dysregulation, and perceived threats could explain aggressive behavior in people with dual diagnoses [152]. For example, anxiety, restricted interests, and CB were positively associated in a sample of people with *Fragile X syndrome* [158]. Finally, mental health could be identified as a mediating factor between victimization history and aggressive behavior in people with IDDs [153].

With regard to sleep problems, although one study found no significant differences [137], the results seemed to suggest significant relationships between sleep disorders and the presence of CB [100,129]. Additionally, two studies revealed psychotropic medication as a risk marker for CB [107,129], specifically self-injurious behavior [107].

Finally, in a sample of people with IDDs and autism, 42% of the variance in CB could be explained by medication, sleep problems, and anxiety [113].

#### 3.3.9. Mood, Interest/Pleasure, Emotional Perception, Emotional Development, and Alexithymia (*n* = 5)

Analysis of these results revealed that CB is predicted by participants’ mood [28,145], although this relationship was significant only in people with IDDs and autism spectrum disorder [145]. The increase in the frequency and severity of self-injurious behavior over time was negatively correlated with interest/pleasure [28]. The emotional perception of people with IDDs was negatively associated with CB, as well as with the difficulty of approach [147]. Additionally, a low level of emotional development was revealed as a risk factor for the appearance of irritability and self-harm [103]. A significant association was established between alexithymia and CB, its severity, and difficulty in its management [4], but this was described by support staff and not by the people exhibiting the CB.

#### 3.3.10. Family, Caregivers, and Support Staff (*n* = 12)

The severity of aggression was positively correlated with the burden on the family caregiver and negatively correlated with uplift [114], and challenging behavior was positively associated with maternal stress [132]. Parental education and family income were revealed as risk factors associated with the occurrence of CB [137].

Perceived stress and emotional exhaustion among support workers was also positively correlated with the occurrence of CB [121,133,149,150]. This association could be mediated by the negative emotions of the support worker [149] or by fear of being attacked [150]. The reactions of support staff to CB were dependent on the type of CB, and negative emotional reactions were positively correlated with CB [128]. Support staff tended to rate the anger of people with IDDs based on overt CB [102].

### 3.4. Intervention Studies (n = 23)

In 23 studies, instruments to assess CB were used to design interventions and explore the effect of these interventions on the CB presented by the participants. The details of the different interventions identified in this review can be found in Table 4.

A total of 1540 people (812 of whom were males) were involved in this category of analysis. Two studies did not report the sex of participants [162,168]. The sample had a mean age of 34.46 years (range = 3–96 years). All participants had IDDs (except 13 participants in one study [138]). Fifteen studies worked with samples of adults [34,35,160,161,163,166,167,168,169,170,171,172,173,177,179], four with samples of children [162,164,176,178], one with adolescents [165], one with adolescents and young people [174], and two were based on combined samples of adults and children [138,175].

CB was considered the dependent variable for evaluation of the effectiveness of the interventions. The most commonly used instrument in this type of study was the ABC [34,35,160,161,162,163,164,165,166,167,168]. The other instruments used were BPI-01 [172,174], CCB [175,176,177], ABC-C [170,171], BPI-PIMD [173], BPI-S [138], ECBI/SESBI-R [178], and MOAS [179], while one study used a combination of two instruments, namely, the ABC and MOAS [102].

The interventions carried out included person-centered active support, positive behavioral support, training of key professionals or home caregivers, and implementation of other specific interventions, such as interventions in residential settings following structured programs. Although a few studies found that the interventions had no significant effects [35,167,170,172], evidence of an intervention was generally reported as reducing the CB of the participants.

Positive behavioral support positively impacted on the reduction in CB [138,171,174], and active person-centered support was used in two studies [35,168]. For example, significant reductions in CB were found after training professionals in active support [168] or after relocating people with IDDs using a decision-making process based on person-centered planning [179]. However, Smith et al. (2002), although reaffirming the effectiveness of active support training for people with IDDs and significant support needs, found no significant changes in CB among people with IDDs. Evidence demonstrated the effectiveness of training key professionals or home caregivers in different skills or intervention procedures in reducing the occurrence of CB [164,166,167,170,175,176,177]. For example, a joint parent–teacher training program [176] and a web-based training program for professionals aimed at improving interaction skills with people with IDDs [177] showed significant positive changes in CB.

Interventions in residential settings following a structured program for people with IDDs and ASD also reported positive trends in the reduction of CB [34,160,161]. For example, Gerber et al. (2011) detected significance in the domains of stereotypic behavior and inappropriate speech. Furthermore, treatment conducted by a team specializing in behavior therapy had significant effects on CB. Significant differences were detected in the overall ABC score and in the lethargy and hyperactivity/noncompliance subscale [163].

With regard to the therapies used, self-centered training or therapy for different levels of IDDs were also explored [162,165,169,173,178]. Positive results in reducing CB were identified in most of the studies. For example, cognitive training that focused on the participants’ attentional and visuospatial skills showed significant improvements on the hyperactivity/noncompliance subscale of the ABC [162]. Additionally, parent–child interaction therapy combined with visual support reduced the CB of a girl with IDD and autism spectrum disorder after the intervention was conducted [178]. However, relaxation activities using a multisensory environment or massage therapy based on the principles of muscle relaxation had no significant effect on problematic behavior [172].

## 4. Discussion

The main aim of this review was to identify the standardized instruments used to assess the CB exhibited by people with IDDs and to analyze their rigor and usefulness. Specifically, we aimed to explore (a) the psychometric characteristics and properties of these instruments, (b) the relationship between the CB assessed and the variables that might be related to it, and (c) the evidence provided by the literature in relation to the psychoeducational interventions carried out based on the results of the assessment of the CB. The results obtained in relation to these objectives attest to the current state of the subject. Our review identified 24 instruments that aimed to assess the CB exhibited by people with IDDs. Moreover, 23 studies explored the psychometric characteristics of some of these instruments using different samples and in different contexts. Similarly, 72 studies examined variables related to CB, and 23 studies considered the results of the different interventions carried out. The results we obtained are set out and discussed below.

First, this review identified 24 instruments that assess and identify different categories and types of CB exhibited by people with IDDs with different support needs throughout their life. Specifically, the validation work included samples ranging from 27 to 3457 participants with IDDs (at different levels) aged from 2 to 84 years. Based on international standards [60,61,62,63,65,66], the instruments showed good psychometric properties that guaranteed the validity and reliability of the results obtained. Sixteen of these instruments analyzed different types of CBs (ABC, ABC-C, ASD-BPA, ASD-PBC, BPI-01, BPI-S, PIMD BPI-PIMD, BPI-S-SCHOOL, CBI, CBQ, CCB, ECBI, SESBI-R, TPBCL, IBR-MOAS, and MOAS) and eight instruments concerned a single type of behavior (A-SHARP, C-SHARP, BSIQ, RBS-R, RBQ, SIT Scale, SBS, and SBS-R). The topographies of the selected behaviors were relevant, even though the dimensions that were used to evaluate them did not appear to be exhaustive [16]. For example, the CBQ [44] contains eight items that assess physical aggression, property destruction, and stereotypic and self-injurious behavior, whereas SBS-R [54] contains twenty-four items to assess stereotypic behavior. Additionally, CB is usually assessed in terms of the frequency and intensity of behaviors. However, some instruments also include other variables such as the duration of the behavior and its impact on the environment. CB needs to be addressed by understanding its nature, and the elements that help us to understand and define the behavior include the context that interacts with the behavior [1,10]. For example, the CBI [52] considers the difficulties in the management of CB, while the SIT Scale [45] qualifies the physical damage produced.

Second, 23 studies, covering nine instruments, continued to explore the psychometric properties of the selected measures or validated them in other populations. The BPI-01 is the most studied instrument [68,69,72,73,74,75,76,77,78,79,80,81,82] and has been translated into several languages [73,74,75,77,78,79]. The results of these studies indicate that the validations carried out had good psychometric properties. The other instruments also validated in different populations include BPI-S [82,83,84,85], RBS-S [86,87,88], ABC [67,68,69], ABC-C [70,71], and MOAS [59].

Third, 72 articles explored the relationship between CB and the variables that can be related to it. The instruments most commonly used to establish this relationship were the ABC, ABC-C, some versions of the BPI-01, or a combination of different instruments. Age [36,98,106,108,151]; the level of adaptive behavior, disability, and language [29,30,68,95,97,99,100,106,107,118,124,129,135,138,145,158]; the presence of autism spectrum disorder [32,95,99,100,103,107,115,116,117,119,120,126,129,141,143,145]; the type of diagnosis linked to a genetic syndrome [101,105,109,111,122,142,145,146,156,158]; psychiatric disorders associated with the severity of the disability [29,96,107,113,125,127,134,139,152,153,154,155,158]; medication and sleep problems [100,107], [113,129]; and the level of stress and emotional exhaustion of family members and caregivers [114,121,128,132,133,137,149,150] were the variables identified as being related to the occurrence of these behaviors. There appears to be robust evidence for some of the variables studied. For example, the presence of autism spectrum disorder appears to have an impact on the presence and occurrence of CB. The support needs and the adaptive behavior of people exhibiting CB also play an important role. The literature clearly indicates that children who need more behavioral support are more likely to exhibit CB [9,180].

Fourth, 23 studies used the identified instruments when designing and implementing behavioral interventions. The ABC [34,35,160,161,162,163,164,165,166,167,168,169] was the instrument most used when conducting these types of studies. The interventions carried out used diverse strategies such as active support, positive behavioral support, training of professionals, environmental changes, and cognitive–behavioral therapy. Positive behavioral support and applied behavioral analysis were the theoretical and practical approaches that underpinned an important part of the interventions. Undoubtedly, there is significant empirical evidence for these systems, and their strategies are considered evidence-based practices [181,182].

This review is not without limitations. First, it is possible that the number of studies analyzed and the breadth of the topics covered influenced the appropriate and precise selection of each of them. Although some strategies were implemented to reduce this issue, such as independent searches in different databases using the names of the instruments as keywords, we should be cautious in the assessment and interpretation of the results. In addition, our method excluded those instruments that assessed other skills, such as social or adaptive behavior, in addition to CB; doing so meant that we did not include all of the instruments that assess CB at some point. However, the identified instruments and the evidence reported in our literature review may help professionals and researchers to take further steps. Specifically, from a professional perspective, many instruments can be used when assessing the CB presented by people with intellectual disabilities. The BPI-01, ABC, and BPI-S appear to be the instruments most commonly used. Their solid psychometric properties, their targeting of more than one type of CB, and their applicability to all populations of people with ID may be some of the reasons for the common use of these instruments. These instruments could help professionals conducting functional assessments to understand behavior and design behavioral interventions [183,184]. Interestingly, little research has been conducted using instruments that focused on one type of CB, such as IBR-MOS, C-SHARP, A-SHARP, or RBS-R. Indeed, people with IDD are likely to present different types of CB based on their behavioral needs [6]. Future research should continue the development and validation of different instruments for the ID population in order to better understand and assess CB [140]. Moreover, understanding CB as the product of dynamic interactions between a complex series of variables that exist within individuals and their social environments [1,185] highlights the need for the development of new methods and systems to evaluate the impact of different personal and contextual variables on the manifestation of CB [186,187]. Research must continue to explore the relationship between different variables (individual and contextual) and the occurrence of CB. As shown in this review, most of the individual variables explored (such as gender, age, and IDD level) focused on the individual. However, the literature suggests that other contextual variables have a significant impact on the treatment of CB. Specifically, training affects professional competence, the emotional well-being of professionals, and the retention of staff in the job [121,188,189]. Moreover, efforts to explore the relationship between CB and other disorders are also needed.

Finally, research should make use of instruments when designing and implementing behavioral interventions. The instruments identified in this review aim to assess CB by understanding the behavior and providing the necessary support for people with IDDs. However, our review only identified 23 studies in this category. Although a high percentage of the studies indicated positive and significant results in reducing CB, relatively few (eight) included a control group. Of these, only one reported fidelity in the implementation of the treatment. Thus, the strategies used and the results obtained must be interpreted with caution, as they lack sufficient empirical validity. Further studies involving solid experimental designs in addition to appropriate replications are recommended.

## 5. Conclusions

The assessment of CB has generated a wide range of instruments to help professionals and researchers better understand these behaviors. Advances in the understanding of the CB exhibited by children, young adults, and adults with IDDs are necessary for the design of better intervention plans that meet their behavioral needs.

Most of the research included in this systematic review focused on the validation of these measures and also on exploring the relationship between the behavior and other variables. Building on advances in this field of knowledge, research should continue to develop new measures based on the latest understanding of CB, as well as exploring the effectiveness of interventions based on assessment results.

## Figures and Tables

**Table 1:** Studies comprising the systematic review (*n* = 23).

Tools (Abbreviation; Reference)	Purpose and Focus	N° Items	Domains	Evaluation Type	Validity and Reliability
ABC or ABC-R ^1^ [36]	Assess the presence and severity of various CB ^2^ Intended for adults and children with IDD ^3^	58	Irritability, agitation, crying; Lethargy, social withdrawal; Stereotypic behavior; Hyperactivity, non-compliance; Inappropriate speech	Frequency, severity	Internal consistency (Cronbach’s α): 0.86 to 0.94 Inter-rater reliability: 0.55 to 0.69 Test-retest reliability: 0.96 to 0.99 Criterion validity: very good
ABC-C ^4^; [37]	Review of the original ABC to remove references to institutional exclusive use Intended for adults and children with IDD	58	Irritability, agitation, crying; Lethargy, social withdrawal; Stereotypic behavior; Hyperactivity, noncompliance; Inappropriate speech	Frequency, severity	Internal consistency (alpha coefficients): 0.84 to 0.94
A-SHARP ^5^ [47]	Measure the severity and direction of aggression Intended for adults with IDD	52	Verbal aggression; Physical aggression; Hostile affect; Covert aggression; Bullying	Severity, ‘‘origin’’ (reactive versus proactive)	Inter-rater reliability (ICCs ^6^) = Problem subscales: 0.59 to 0.78. Provocation subscales: 0.54 to 0.78 Internal consistency (item-total correlations): Verbal aggression: 0.54 to 0.89 Concurrent validity with BPI (Pearson correlations): 0.33 to 0.86
C-SHARP ^7^ [41]	It examines different forms of aggression and categorizes the child’s behavior as reactive or proactive Intended for children with IDD	52	Verbal aggression; Bullying; Covert aggression; Hostility; Physical aggression	Severity, the ‘‘origin’’	Inter-rater reliability (ICCs) = Problem subscales: high. 0.67 to 0.91; Provocation Scale: 0.01 to 0.76 Internal consistency (Cronbach’s α): 0.74 to 0.90 Item-total correlations: 0.59 to 0.75
ASD-BPA ^8^ [49]	Part of a comprehensive assessment for adults with ASD ^9^ and PDD-NOS ^10^, along with the diagnosis and comorbidity Intended for adults with ASD and IDD	19	Aggression/destruction; Disruptive behavior; Self-injurious behavior	Occurrence	Inter-rater reliability (Kappa coefficients): 0.14 to 0.68 Test-retest reliability (Kappa coefficients): 0.24 to 0.81 Internal consistency (KR-20 coefficients): 0.43 to 0.83
ASD-PBC ^11^ [48]	Part of a comprehensive battery of measures that assess CBs, comorbid psychopathology and ASD symptoms Intended for children and adolescents with ASD with IDD	18	Internalizing scale (Self-injurious behavior, stereotyped behavior, inappropriate sexual behavior, and other odd behaviors) and Externalizing scale (physical and verbal aggression, property destruction, and tantrums)	Occurrence, severity	Inter-rater reliability: 0.49; mean agreement of 92% Test–retest reliability: 64 with mean agreement at 92% Inter-item correlation: 0.32 Internal consistency: 0.90
BPI-01 ^12^ [55]	Behavior rating scale Intended for adults and children with IDD	52	Self-injurious behavior; Stereotyped behavior; Aggressive/ destructive behavior	Frequency; severity	Inter-rater reliability (ICCs): 0.91 Internal Consistency (Cronbach’s α): 0.83 Factor Structure (RMSEA): 0.078
BPI-S ^13^ [56]	Abbreviated version of the BPI-01 Intended for adults and children with IDD	30	Self-injurious behavior; Stereotyped behavior; Aggressive/destructive Behavior	Frequency; severity	Internal consistency (Cronbach’s alpha) 0.85 to 0.87 Convergent validity: NCBRF, ICAP, DASH-II, ABC Factor Structure: models fit the data well Spearman r correlations between BPI-01 and the BPI-s: very high (r 0.958 to 0.99)
BPI-PIMD ^14^ [53]	Revised version of BPI-01 Dutch with some adaptations to increase its applicability to people with PIMD ^15^ Intended for people with PIMD	58	Self-injurious behavior; Stereotypical behavior; Withdrawn behavior; Aggressive/destructive behavior	Frequency; Severity	Test–retest reliability: frequency scale good to excellent Internal consistency (Cronbach’s α): 0.85
BPI-S-SCHOOL ^16^ [51]	Adaptation of the BPI-S for children with IDD in the school environment Intended for children and young people with IDD	32	Self-injurious behavior; Stereotyped behavior; Aggressive/destructive behavior	Frequency; Severity	Internal consistency (Cronbach’s α): good to excellent
BSIQ ^17^ [42]	Continuous dimensional instrument that assesses repetitive behaviors, restricted interests and other unusual sensory behaviors Intended for children and adults with range of neurodevelopmental disorders, including ASD	174	Repetitive Sensory Motor behaviors; Insistence on Sameness; and others	Type; Frequency; Intensity; Age of onset; Duration; Sensory interests	Inter-rater reliability: stable Item-total correlations 0.20 to 0.51 (Repetitive Sensory Motor) and 0.12 to 0.53 (Insistence on Sameness) Internal consistency (Cronbach’s α): 0.831 (Repetitive Sensory Motor) and 0.731 (Insistence on Sameness)
CBI ^18^ [52]	Assess the severity of the CB Intended for children and adults with IDD	19	Self-injury, physical aggression, verbal aggression, disruption of the environment and inappropriate vocalizations	Occurrence; Frequency; Duration; Management strategies used by carers.	Inter-rater: 0.90 Test–retest agreement: 0.96 Concurrent validity: ABC (0.19 and 0.68)
CBQ ^19^ [44]	Assessment of CB Intended for people with IDD	8	Self-injurious behavior; Physical aggression; Property destruction; Stereotypic behavior	Prevalence and topography	Inter-rater reliability: good 0.46 to 0.72
CCB ^20^ [43]	Assess aggressive behavior and other CB Intended for children and adults with IDD	Part 1 (14); part 2 (18)	Part 1: Aggressive behaviors involving harmful, physical contact with others; Part 2: A list of other types of challenging behavior	Frequency; Severity; Management difficulty	The rating scales are not sufficiently reliable as measures of change especially at the individual level
ECBI ^21^ [40]	A companion behavioral rating scale for children from a range of ethnic and socioeconomic, chronically ill and with IDD	36	Noncompliance; Defiance; Aggressiveness; Impulsiveness	Intensity	Internal consistencies: 0.93 to 0.98
SESBI-R ^22^ [40]	A companion behavioral rating scale for children from a range of ethnic and socioeconomic, chronically ill and with IDD	38	Noncompliance; Defiance; Aggressiveness; Impulsiveness	Intensity	Inter-rater: 0.43 to 0.84 Test retest: Intensity 0.81, Problem 0.84 Internal consistency (Chronbach’s α): Intensity 0.98
IBR-MOAS ^23^ [39]	Measure of aggressiveness in people with IDD Intended for adults with IDD	20	Verbal aggression toward others; Physical aggression against other people; Physical aggression against objects; Physical aggression against self; and Verbal aggression toward self	Frequency; Severity	Inter-rater reliability (Cronbach’s α): 0.70 to 0.83 Test–retest reliability (Cronbach’s α): 0.84 to 0.96
MOAS ^24^ [46]	Measure nature and prevalence of types of aggression It has been used with psychiatric populations and adults with IDD	20	Verbal aggression; Aggression against property; Autoaggressions; Physical aggressions	Severity	Inter-rater reliability (Pearson r): 0.85 to 0.94 Test-retest: 0.72 Internal consistency: (coefficient of concordance) W = 0.68
PBCL ^25^ [58]	Short scale to assess CBs Intended for children and adults with IDD	28	Personal violence; Violence against property; Self-harm; Sexually inappropriate; Contrary; Demanding; Disappearing behavior	Severity	Inter-rater reliability (kappa): 0.91; 95% CI 0.83–0.99
RBQ ^26^ [50]	Assessment of the nature of repetitive behavior in people with different neurological syndrome Intended for children and adults with IDD	19	Stereotyped behavior; Compulsive behavior; Insistence on sameness; Restricted preferences; Repetitive speech	Frequency	Inter-rater reliability (Spearman coefficients): 0.46 to 0.80 at item level Test retest reliability (Spearman coefficients): 0.61 to 0.93 Internal consistency: (α > 0.80)
RBS-R ^27^ [38]	Evaluate ritualized behaviors, insistence on equality, and restricted interests Intended children and adults with ASD and/or IDD	43	Stereotyped Behavior; Self-Injurious Behavior; Compulsive Behavior; Ritualistic Behavior; Sameness Behavior; Restricted Behavior	Severity	Inter-rater reliability: 0.55 to 0.78 Test-retest reliability: 0.52 to 0.96
SIT Scale ^28^ [45]	Measure to quantify surface tissue damage caused by self-injurious behavior Individuals with functional abilities varied	3 parts	SIB	Topography; location of the injury in the body; type of injury; number of injuries; severity.	Part 2: Reliability: Mean = 97%; Median = 98% R (86–100) Part 3: Number Index(NI) = 90%; Severity Index(SI) = 92%; Estimate of Current Risk = 100%
SBS ^29^ [57]	Assess stereotyped behavior in people with IDD Intended for adolescents and adults with IDD	26	Stereotyped	Frequency	Test-retest reliability (ICC) 0.82 Inter-rater reliability (ICC) 0.33 Internal consistency: 0.88
SBS-R ^30^ [54]	SBS review Intended for adults with IDD	24	Stereotyped	Severity; Frequency	Test-retest Reliability (ICC): 0.93 and 0.71 Inter-rater agreement (ICC): 0.76 and 0.75 Internal consistency: 0.91 Criterion validity with ABC-R “Stereotypy”

Note: ^1^ Aberrant behavior Checklist; ^2^ Challenging behavior; ^3^ Intellectual and developmental disabilities; ^4^ Aberrant behavior Checklist-Community; ^5^ Adult Scale of Hostility and Aggression: Reactive–Proactive; ^6^ Intraclass correlation coefficient; ^7^ Children’s Scale of Hostility and Aggression: Reactive–Proactive; ^8^ Autism Spectrum Disorders—Behavior Problems for Adults; ^9^ Autism Spectrum Disorder; ^10^ Pervasive developmental disorder—not otherwise specified; ^11^ Autism Spectrum Disorders—Problem Behaviors Child; ^12^ The Behavior Problems Inventory; ^13^ The Behavior Problems Inventory—Short; ^14^ The Behavior Problems Inventory for people with PIMD; ^15^ profound intellectual and multiple disabilities; ^16^ The Behavior Problems Inventory for School; ^17^ Behavior and Sensory Interests Questionnaire; ^18^ Challenging Behavior Interview; ^19^ Challenging Behavior Questionnaire; ^20^ Checklist of Challenging Behavior; ^21^ Eyberg Child Behavior Inventory; ^22^ Sutter–Eyberg Student Behavior Inventory—Revised; ^23^ Institute for Basic Research—Modified Overt Aggression Scale; ^24^ Modified Overt Aggression Scale; ^25^ The Problem Behavior CheckList; ^26^ The Repetitive Behavior Questionnaire; ^27^ The Repetitive Behavior Scale—Revised; ^28^ Self-Injury Trauma Scale; ^29^ The Stereotyped Behavior Scale; ^30^ The Stereotyped Behavior Scale Revised.

**Table 2:** Measurement studies of challenging behavior instruments.

Tool	Study	Country	N (Male)	Range	Psychometric Properties
ABC	[67]	Norway, English	339 (220)	4–18	Internal consistency (Cronbach’s α): 0.76 to 0.95
ABC/BPI-01/RBS-R	[68]	EEUU and Peru; English	180 (110)	0.33–4	BPI-01 Test-retest (Spearman’s ρ correlations): 0.41 to 0.64; ICC ^1^ = 0.68 to 0.80 Internal consistency (Cronbach’s α): 0.85 to 0.90 Convergent with ABC and RBS-R ABC Internal consistency (Cronbach’s α): 0.96 RSB-R Internal consistency (Cronbach’s α): 0.89
ABC/BPI-01	[69]	UK; English	69 (58)	9.3–29.59	Convergent validity ABC with BPI-01 (MANCOVA and Multiple regression)
ABC-C	[70]	EEUU English	601 (339)	6–22	Internal consistency (Cronbach’s α): 0.77 to 0.95 Construct validity: four factor accounted 48% of the total common variance
ABC-C	[71]	Austria and German, English	270 (151)	18–80	Inter-rater: 0.79 Test-retest (Spearman’s ρ correlations): 0.97 (4 weeks) and 0.43 (2 years) Internal consistency (Cronbach’s α): 0.95 Factor validity: Five factors
A-SHARP BPI-01	[72]	EEUU, English	155 (108)	16–71	Internal consistency (Cronbach’s α): 0.95 (problem); 0.90 (provocation) Congruent validity with the BPI-01 Aggressive/Destructive Behavior (Pearson correlations): 0.15 to 0.71 Construct validity: five factor ^1^ χ^2^ (1.070) = 1494.07, *p* < 0.001, ^2^ CFI = 0.949, ^3^ TLI = 0.946, ^4^ RMSEA = 0.051
BPI-01	[73]	China English	222 (167)	1.5–21.5	Internal consistency (Cronbach’s α): 0.92 Construct validity: Three factors model fit the data well
BPI-01	[74]	Brazil, English	60 (38)	6–18	Internal consistency subscales (Cronbach’s α): 0.65 to 0.91 Convergent validity with ^5^ ASQ and ^6^ CBCL/6–18
BPI-01	[75]	Holland, English	195 (113)	2–73	Inter-rater: ^7^ ICC = 0.73; ^8^ EA/AA = 83.6%/90.1%; Cohen’s kappa: 0.36 Intra-rater ICC = 0.93; EA/AA = 89.6%/95.4%; Cohen’s kappa: 0.63 Internal consistency (Cronbach’s α): 0.89 Construct validity: Three factors model fit the data well Convergent validity with ABC
BPI-01	[76]	EEUU, English	425 (235)	15–87	Internal consistency (Cronbach’s α): frequency and severity samples 1 and 2. Stereotyped Behavior and Aggression/Destruction subscales 0.65 to 0.87 and SIB 0.40 to 0.48 Inter-rater: ^9^ SIB frequency 0.67, severity 0.63; Stereotyped Behavior frequency 0.41, severity 0.50; Aggression/Destruction frequency 0.80, severity 0.77 Test–retest: SIB frequency 0.65, severity 0.70; Stereotyped Behavior frequency 0.45, severity 0.28; Aggression/Destruction frequency 0.66, severity 0.67 Construct validity: Three factors, RMSEA = frequency 0.063 and severity 0.075
BPI-01	[77]	Korea, English	52 (31)	3–36	Content validity: nine questions on content validity Inter-rater: ICC = frequency 0.72 and severity 0.070 Test–retest: ICC = frequency 0.87 and severity 0.84 Internal consistency (Cronbach’s α): frequency 0.88 and severity 0.87
BPI-01	[78]	Sweden, English	915 (503)	18–87	Internal consistency (Cronbach’s α): frequency 0.84 and severity 0.85 Construct validity: Three factors χ^2^ = 3832.7, df = 1124, χ^2^/df = 3.41, *p* < 0.001 and RMSEA = 0.051,
BPI-01	[79]	EEUU, English	115 (51)	3–23	Internal Consistency (Cronbach’s α): 0.88 to 0.95 Convergent validity with ^10^ NCBRF
BPI-01	[80]	EEUU, English	237 (160)	4–22	Inter-rater (teacher-teacher): ICC = 0.76 Inter-rater (teacher-parent): ICC = 0.24 Test-retest: ICC = 0.84 Internal consistency: 0.59 to 0.88 Convergent validity with NCBRF Construct validity: Three factors, RMSEA = frequency 0.083 CFI = frequency 0.52
BPI-01	[81]	EEUU, English	130 (92)	Adults	Inter-rater: ICC = 0.75 to 0.84 Test–retest: ICC = 0.88 to 0.91 Internal consistency (Cronbach’s a): 0.61 to 0.90 Convergent validity with ^11^ ICAP
BPI-01/BPI-S	[82]	USA, United Kingdom, Romania, Holland English	1122 (768)	2.1–93	Construct validity: Three factors BPI-01: χ^2^ (260) = 730.92, *p* < 0.05; CFI = 0.93; TLI = 0.98; RMSEA = 0.04 BPI-S: χ^2^ (260) = 860.15, *p* < 0.05 CFI = 0.93 TLI = 0.97; RMSEA = 0.05
BPI-S	[83]	Japan, English	227 (142)	adolescents/adults	Test–retest: ICC = frequency 0.954; severity 0.927 Inter-rater: ICC = frequency 0.721; severity 0.740 Internal consistency (Cronbach’s a): frequency 0.83; severity 0.83 Criterion-related validity (Spearman correlation) with ^12^ CDSPB r = 0.499 and ^13^ ABC-J r = 0.699
BPI-S	[84]	EEUU, English	232 (157)	16–71	Inter-rater: ICC = 0.46 to 0.74 Test–retest r (Pearson’s r correlation): Primary raters 0.79 to 0.91 and secondary raters 0.66 to 0.84 Internal Consistency (Cronbach’s α): 0.91 Construct validity: Three factors. χ^2^ (402) = 2018.8, *p* < 0.001; CFI = 0.74, TLI = 0.72; ^14^ SRMS = 0.08; RMSEA = 0.08
BPI-S	[85]	France, English	305 (172)	7–24	Inter-rater ICC = frequency 0.66 to 0.81 and severity 0.54 to 0.92 Test retest: (Pearson correlation coefficients): frequency 0.45 to 0.53 and severity positive and significant covariance between aggressive and destructive behaviors and SIB Internal consistency (Cronbach’s α): frequency 0.90 and severity 0.62 Construct validity: Three factors. Frequency CFI = 0.96; TLI = 0.96 and RMSEA = 0.07. Severity CFI = 0.91; TLI = 0.90; RMSEA = 0.04
MOAS	[59]	UK, English	14 (9)	23–58	Inter-rater: ICC = 0.93
RBS-R	[86]	Japan, english	310 (243)	3–40	Internal consistency (Cronbach’s α): 0.928
RBS-R	[87]	Germany, English	948 (546)	4–17	Internal consistency (Cronbach’s a): 0.96 Concurrent-Discriminant Validity with ABC (r = 0.69); CBCL (r = 0.72) and ^15^ SRS (r = 0.70) Construct validity: four-factor solution
RBS-R	[88]	Spain, English	233 (181)	3–63	Inter-rater: ICC= Test-retest: ICC = 0.97 Item-total correlations 0.50 and 0.80 Internal consistency (Cronbach’s α): 0.93 Concurrent-divergent validity with ^16^ SCQ-B (r = 0.42–0.68)

Note: ^1^ chi-square:χ^2^; CFI: ^2^ Comparative Fit Index; ^3^ TLI: Tucker–Lewis index; ^4^ RMSEA: Mean Square Error of Approximation; ^5^ ASQ: Autism Screening Questionnaire; ^6^ CBCL/6–18: Child Behavior Checklist for Ages 6–18; ^7^ ICC: Intraclass correlation coefficient; ^8^ EA/AA: Exact agreement/adjacent agreement; ^9^ Self-injury behavior; ^10^ NCBRF: Nisonger Child Behavior Rating Form [89]; ^11^ ICAP: Inventory for Client and Agency Planning [90]; ^12^ CDSPB: Criteria for Determining Severe Problem Behavior; ^13^ ABC-J: Aberrant Behavior Checklist—Japanese [91]; ^14^ SRMS: standardized root mean square residual; ^15^ SRS: The German version of Social Responsiveness Scale [92]; ^16^ SCQ: Social Communication Questionnaire, SCQ form B [93].

**Table 3:** Relational studies using the instruments identified in the review.

Tool	Study	*n* (Male)	Range	Variables Related to Challenging Behavior	Variables Not Related to Challenging Behavior
ABC	[94]	82 (46)	11–17	Age	Age (General pattern stability)
ABC	[95]	818 (433)	18–90	Adaptive behavior; ASD ^1^ symptoms	Gender
ABC	[96]	312 (134)	-	Psychiatric disorder	*np* ^2^
ABC	[97]	427 (250)	15–86	Adaptive behavior; social, community and home participation; support	*np*
ABC	[98]	132 (89)	16–66	Age	*np*
ABC	[99]	140 (73)	14–72	Level IDD ^3^; ASD symptoms; quality of life	*np*
ABC	[100]	106 (90)	≥18 years	Age; language impairment; level IDD; sleep disorder; ASD symptoms; gastrointestinal disorder	Age
ABC	[101]	122 (67)	1–35	Developmental and Epileptic Encephalopathies	*np*
ABC/MOAS	[102]	181 (128)	28–47	Staff rate users’ anger	*np*
ABC/MOAS	[103]	203 (139)	≥18 years	Emotional development, ASD symptoms	*np*
ABC/SIT Scale	[104]	32 (26)	0.67–4.3	Age	*np*
ABC-2	[105]	62 (34)	2.5–18	Dup15q syndrome	*np*
ABC-C	[106]	240 (156)	≥18 years	Age, adaptive behavior	Epilepsy
ABC-C	[107]	833 (432)	18–84	Age; level IDD; psychiatric disorder; ASD symptoms; psychiatric medication; pain	Epilepsy; sex
ABC-C	[108]	124 (64)	2–26	Age; gender	Age
ABC-C	[109]	34 (20)	≥18 years	DS ^5^ and dementia	*np*
ABC-C	[110]	80 (39)	16–68	Negative life events	*np*
ABC-C	[33]	97 (73)	0.80–4.81	Age	*np*
ABC-C	[111]	60 (30)	0.92–23	Phelan-McDermid syndrome	*np*
ABC-C/CBI	[112]	100 (48)	Children	* np *	Cornelia de Lange Syndrome
ABC-C/MOAS	[113]	167 (137)	5–18	Sleep disorder; psychiatric disorders	*np*
ABC-C/MOAS	[114]	44 (31)	19–56	Family caregiver burden; family caregiver uplift	*np*
ASD-BPA	[115]	175 (94)	20–87	ASD symptomatology	Race
ASD-BPA	[116]	298 (167)	21–88	ASD symptoms	*np*
ASD-BPA	[117]	100 (72)	29–72	ASD symptoms; epilepsy	*np*
ASD-BPA	[118]	45 (23)	16–88	Language impairment; ASD symptoms	*np*
ASD-BPA	[119]	70	18–43	Quality of life mediated the relationships between ASD- challenging behaviors	*np*
ASD-PBC/BPI-01/RBS-R	[120]	313 (211)	2–17	ASD symptoms	*np*
A-SHARP	[121]	200 (109)	≥18 years	Burnout and instability of support staff	*np*
BPI-01	[122]	99 (43)	1.5–51	Smith–Magenis Syndrome	*np*
BPI-01	[123]	1871 (1085)	2.1–93	Stereotyped behavior construct–self injury construct	*np*
BPI-01	[124]	120 (58)	≥18 years	Level IDD	*np*
BPI-01	[29]	244 (156)	10–19	Level IDD; psychiatric disorders	*np*
BPI-01	[125]	159 (69)	19–56	Psychiatric disorders	*np*
BPI-01	[126]	57 (39)	4.25–18	ASD symptoms; DS ^4^	*np*
BPI-01	[127]	39 (22)	19–49	Psychiatric disorders	*np*
BPI-01	[128]	51 (31)	8–70	Emotional reactions support staff	*np*
BPI-01	[129]	915 (504)	18–87	Language impairment; level IDD; sleep disorder; sensory hypersensitivity; psychiatric medication; DS ^5^ and cerebral palsy; ASD symptoms	*np*
BPI-01	[130]	95 (59)	15–86	Reliability of high-rate versus low-rate responses.	*np*
BPI-01	[131]	157 (130)	3–14.2	* np *	Age; gender
BPI-01	[31]	180 (76)	1.5–61.4	Language impairment; level IDD	Level IDD
BPI-01	[132]	46 (31)	4–27	Maternal stress	*np*
BPI-01	[133]	Staff’s data	Staff’s data	Perceived stress and emotional exhaustion of support staff	*np*
BPI-01	[134]	58 (39)	21–60	Psychiatric disorders	*np*
BPI-01	[135]	189 (111)	18.3–85.9	Level IDD	Epilepsy
BPI-01	[136]	30 (20)	2–65	Intervention plans	*np*
BPI-01	[137]	180 (112)	0.33–4	Age; gender; diagnosis; communication levels; visual impairment; parent education; family income	Sleep disorder; psychiatric medication
BPI-01/ASD-BPA	[32]	57 (38)	23–81	ASD symptoms	*np*
BPI-S	[138]	265 (134)	≥18 years	Language impairment; level IDD	*np*
BPI-S	[139]	160 (88)	18–71	Psychiatric disorders	*np*
BPI-S	[140]	598 (266)	≥18 years	Negative life events	*np*
BPI-S	[141]	129 (100)	3–18	Age of ASD diagnosis	*np*
BPI-S	[142]	22 (11)	0.75–28	Phenotype of Pallister–Killian Syndrome	*np*
BPI-S/CBI	[28]	50 (38)	19–49	Interest/pleasure; negative mood	*np*
BPI-S/C-SHARP	[143]	305 (172)	7–18	ASD and age	*np*
CBQ/RBQ	[144]	441 (337)	4–15,9	* np *	Tuberous sclerosis complex
CBQ/RBQ	[145]	321 (276)	4–62	DS ^5^ and FXS ^5^; level IDD; negative mood; ASD symptoms associated with genetic syndrome	*np*
CBQ/RBQ	[146]	79 (42)	≥18 years	Tuberous sclerosis complex	*np*
CCB	[147]	96 (50)	18–79	Emotional perception skills	*np*
CCB	[148]	96 (50)	18–79	Alexithymia	*np*
CCB	[149]	Staff’s data	Staff’s data	Burnout and cognitive variables support workers	*np*
CCB	[150]	Staff’s data	Staff’s data	Burnout and fear of assault support workers	*np*
CCB	[151]	53 (21)	42–92	Age	Age
CCB	[30]	74 (49)	19–73	Level IDD	*np*
IBR-MOAS	[152]	4069 (2441)	≥18 years	Psychiatric disorders	*np*
MOAS	[153]	215 (118)	18–65	Negative life events; psychiatric disorder	*np*
MOAS	[154]	296 (162)	18–65	Psychiatric disorder	*np*
MOAS	[155]	296 (162)	18–65	Psychiatric disorder	*np*
RBQ	[156]	797 (519)	4–51	Genetic syndromes	*np*
RBS-R	[157]	49	0.6–6.75	Age; typical developmental	Gender
RBS-R	[158]	39	6–10	SXF; level IDD; psychiatric disorder	*np*
RBS-R	[159]	61 (61)	3–5	*np*	FXS; ASD

Note: ^1^ Autism Spectrum Disorders; ^2^ not provided; ^3^ Intellectual and developmental disabilities; ^4^ Down’s Syndrome; ^5^ *Fragile X syndrome*.

**Table 4:** Intervention studies included in the review.

Tool	Study	n (Male)	Age (Range)	IDD ^1^ Level (Mild/Moderate/Severe/Profound)	Design	Components	Outcomes
ABC	[160]	19 (15)	39 (*np* ^2^)	*np*	Longitudinal prospective design Experimental design	Autism Programme with a Structured Method	No significant changes in experimental group Significant reduction in social withdrawal behavior in the control group
ABC	[34]	30 (23)	39.9 (24–62)	0/4/19/7	Quasi-experimental, repeated-measures design	Autism Programme with a Structured Method	A significant reduction in behavior disorders
ABC	[161]	31 (23)	39.7 (24–62)	0/4/20/7	Quasi-experimental between-groups	Autism Programme with a Structured Method	Stereotypic behavior and inappropriate speech significantly decreased
ABC	[162]	200 (*np*)	*np* (6–13.11)	Mild-moderate	Experimental Design between-group	Cognitive remediation	Significant reduction in CBs ^3^
ABC	[163]	63 (37)	40.45 (*np*)	Mild-profound	Parallel-group, randomized, single-blind controlled trial	Community-based specialist behavior therapy	Significant differences in total scores
ABC	[164]	8 (5)	Children	0/0/8/0	Quasi-experimental, repeated-measures design	A functional assessment based consultation in cooperation with a team of teacher	Improved score on behavior scales
ABC	[165]	19 (16)	16.58 (12–20)	0/0/19/0	Experimental Design between-group	Imitation training	Moderate to large effects on CB
ABC	[166]	11 (7)	47.45 (*np*)	0/4/6/1	One group pretest–post-test design with a double pretest	PBS training for staff in reducing CBs of individuals with IDD	Significant reductions in CB
ABC	[167]	113 (83)	34.6 (*np*)	*np*	A multicentre, two-arm cluster randomized controlled trial (PBS/TAU)	PBS training for staff	Did not reduce challenging behavior in people with IDD and comorbid ASD ^4^
ABC	[168]	29 (*np*)	44 (20–61)	Severe-profound	Quasi-experimental, repeated-measures design	Person-Centred Active Support	Significant reduction in CB
ABC	[35]	188 (105)	67 (20–79)	*np*	Quasi-experimental between-groups	Person-Centred Active Support	The effectiveness of support offered to people with CB did not significantly increase
ABC-(H/I)/MOAS	[169]	181 (128)	37.75 (27.5–48.5)	Mild-moderate	Multicentre cluster randomized controlled trial (RCT)	Cognitive–behavioral therapy	Keyworkers and home carers showed significantly better outcomes at 16-week follow-up
ABC-C	[170]	245 (157)	37 (25–51)	41/76/127/0	Multicentre cluster researcher-masked randomized controlled trial	Staff training in Positive Behavior Support	No treatment effects were found
ABC-C	[171]	81 (35)	39.7 (19–84)	*np*	Experimental Design between-group	Positive Behavior Support	Significant reductions in CB
BPI-01	[172]	42 (17)	43.40 (18–64)	Severe-profound	Experimental Design between-group	Relaxation activities: multisensory environment and massage therapy	No significant differences in frequency and severity
BPI-S	[138]	85 (27)	25.38 (3–73)	*np*	Quasi-experimental, repeated-measures design	Positive Behavior Support	Significant reductions in CB
BPI.PIMDI	[173]	15 (8)	43.33 (18–55)	Severe-profound	Quasi-experimental, repeated-measures design	Soundscapes (an application for smartphones)	Significant reduction in the severity of stereotyped behavior
BPI-01	[174]	32 (24)	21 (17–29)	0/11/21/0	Longitudinal Quasi-experimental, repeated-measures design	Positive Behavior Support + Systemic approaches. Community intensive servie for adults with IDD and challenge behaviors: The Southwark Enhanced Intervention Service	Improvements in behavior, well-being, quality of life and financial savings
CCB	[175]	60 (36)	35.5 (3–70)	12/21/21/16	Experimental Design between-group A non-randomized matched control group design	Person- focused training	Reductions in the frequency, management difficulty and severity of CB
CCB	[176]	37 (29)	9.5 (*np*)	Severe-moderate	A within-subjects, pre- and post-, quasi-experimental, repeated-measures design	Training program delivered at the same time to teaching staff and family careers	Significant positive changes were found regarding ratings of CB
CCB	[177]	1 (1)	40	0/0/1/0	A single subject experimental design	Web-based training program aimed at improving careers’ abilities to interact with people with IDD	Significant reduction in CB
ECBI/ SESBI-R	[178]	1 (0)	5	0/1/0/0	Case study pre–post-intervention	Parent–Child Interaction Therapy combined with visual supports	Significant reduction in CB
MOAS	[179]	49 (36)	50.05 (31–96)	1/3/11/34	Quasi-experimental, repeated-measures design	Community resettlement using a person-centred approach	All areas of aggressive defiant behavior were significantly reduced

Note: ^1^ Intellectual and developmental disabilities; ^2^ Not provided; ^3^ challenging behavior; ^4^ Autism Spectrum Disorder.

**Figure A1:**
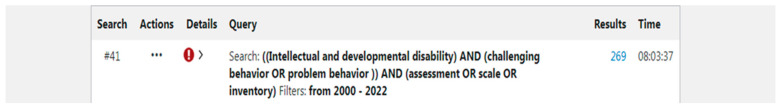
Search Strategy through Electronic Databases.

## Data Availability

Not applicable.
